# Temporary migration of Romanian Roma people to European countries

**DOI:** 10.3389/fsoc.2025.1577497

**Published:** 2025-06-04

**Authors:** Luiza Meseşan-Schmitz, Claudiu Coman, Diana-Cristina Bódi, Mihaela Gotea

**Affiliations:** Faculty of Sociology and Communication, Department of Social Sciences and Communication, Transilvania University of Brasov, Brasov, Romania

**Keywords:** Roma population, discrimination, temporary migration, return migration, push-pull factors, Romania

## Abstract

**Introduction:**

Roma people in Europe are still in a great risk of social exclusion because of the stereotypes, prejudices, and discrimination against them, known as Antigypsyism. They also encounter high levels of poverty, lower levels of education, housing conditions, and health care, high rates of unemployment, and so on. Based on the push-pull theory, the present study examines the phenomenon of international migration of the Roma population from Olt County, Romania, capturing the specific factors that led to their migration and return to the country, and also the effects of this phenomenon on the community at the place of origin.

**Methods:**

our study used a mixed-methods approach, applying a non-standardized questionnaire to 796 Roma people who have experienced international, temporary migration and currently live in Olt County from Romania and semistructured interviews with 15 managers and representatives of the local public and socio-cultural institutions from the same region.

**Results:**

the results show us that the main push-pull factors of external migration of Roma people from Romania, as well as of their return home are economic and socio-cultural ones. Our data can add to the mentioned theory new pull factors for migration to certain countries, such as the friendly climate and easier learning of the language of the host country. We have also discovered that family is the main factor for returning home to Romania, and also the disappointment of their migration experience. The effects of their return migration on them and the community can be positive (e.g., cultural exchange, awareness of the role of education), but also negative (e.g., increasing unemployment, the negative image of Romania).

**Conclusion:**

the findings highlight some assumptions of the push-pull theory, but they also bring new perspectives for understanding and approaching this phenomenon. The perspectives of Roma and representatives of institutions are different regarding the push factors that generate external migration of Roma, Roma identify only economic factors that lead to migration, while managers and representatives also talk about socio-cultural factors involved in the decision to migrate of Roma. The study also identifies the implications generated by the return home of the Roma, with economic, socio-cultural, and educational effects, but also effects at the level of public policies. We believe that the push-pull factors of external migration and the effects of Roma's return to the country are interconnected, generating an amplification of the problems for which Roma migrate. Therefore, they constitute solid arguments for building and streamlining social integration policies for Roma.

## 1 Introduction

According to the European Parliament (2024), the Romani people represent Europe's largest ethnic minority. It is estimated that there are 10–12 million in Europe, and about 6 million Roma people live in the EU, most holding EU citizenship. The estimated share of Roma people in the various member states ranges from 30% in Romania, 12.2% in Bulgaria, 12.2% in Hungary, 12.2% in Spain, 7.9% in Slovakia, 6.5% in France, 3.2% in Czechia, 2.8% in Greece, 2.4% in Italy, 1.7% in Germany to less than 1% in most of the other EU member states. According to 2019 data, Roma people tend to be younger (25.1 years old) than the EU population average (40.2 years old).

It is one of the most disadvantaged minorities. Romani people in Europe are constantly denied their rights to housing, health care, education, and work (Amnesty International, 2020). Excessive force, police brutality, and misconduct against Romani people continue to be reported across the EU, in line with the findings of the FRA - European Union Agency for Fundamental Rights (2022).

The European Union Agency for Fundamental Rights (FRA) report (2022) also revealed that Romani people are subject to widespread poverty, inadequate living conditions, poor health, exclusion from the labor market, and harassment. This report (FRA - European Union Agency for Fundamental Rights, 2022, p. 108) underlines that: “In 2021, the fundamental rights of Roma and Travelers are still not fully respected. Antigypsyism, discrimination, poverty, and social exclusion, as well as hate crime and hate speech, continue to affect a disproportionate number of Roma and Travelers across the EU”.

Employment is vital for individuals' societal integration, but access to the labor market is still low for the Roma population in Europe. The EU-MIDIS II report (FRA - European Union Agency for Fundamental Rights, 2017) finds that only one in four Roma aged 16 or over were “employed” or “self-employed” at the time of the survey. Roma women reported much lower employment rates, 16% compared to Roma men, 34%. Working abroad was identified as a source of income to a great extent in 2017.

The European Union Agency for Fundamental Rights has conducted the Roma Survey 2021 on 87% of the estimated Roma population in the EU or 53% of the estimated Roma population in Europe. The main results demonstrate that exclusion, deprivation, discrimination, and racism remain the reality for too many of Europe's Roma in their daily lives (FRA - European Union Agency for Fundamental Rights, 2023). Often, in society, the Roma are seen as a threat to the population and to the state itself, due to the perception that they “have a natural predisposition to commit criminal acts”, which emphasizes the idea that the Roma must be controlled, to always be kept under surveillance (Fejzula, 2019).

An anti-Roma prejudice has a specific feature compared to other types of ethnic prejudice, it is characterized by the image of Roma as being law-breaking and lazy people who do not deserve any state benefits (Sam Nariman et al., 2020). For example, in Italy, Roma people are perceived as being untrustworthy individuals involved in illicit and criminal activities, engaged in anti-social behaviors that are a “burden on society” (Villano et al., 2017) or Cousin et al. (2021) showed that their members suffer from a univalent prejudice from Italians that is characterized by stereotypical traits such as being deceitful, criminal, cunning, dirty, suspicious and dangerous. The negative stereotypes of Roma can serve as motivational justifications for their moral exclusion, as Hadarics and Kende (2019) found in Hungary. This moral exclusion means that there is a dividing line that separates'moral' individuals – those who can be part of and fully participate in society – from'immoral' ones, frequently blamed and held responsible for their situation. Negative stereotypes about the group allow perceivers to justify any discriminatory, unfair, or immoral behavior toward the out-group. Romanians' racism toward Roma is illustrated by Dolea and Suciu (2024). In a study on the content of stereotypes in Romania, Roma people were included in a cluster characterized by low warmth and low competence (alongside drug addicts, delinquents, and politicians). This cluster had stable memberships across different regions of the country. There is a clear delimitation between Roma people and the rest of the social groups; the evaluation of Roma people seems to have negative connotations that are culture-specific (Stanciu et al., 2017).

Recognizing feelings toward the out-group is also crucial for understanding and examining intergroup dynamics. In their study, Colledani et al. (2018) investigated how Dark Triad traits correlate with emotions and tendencies of approach/avoidance toward the Roma people in Italy. Their results indicated that dark traits influence intergroup dynamics, and their connections with approach/avoidance tendencies are influenced by emotional reactions. Among the traits, Machiavellianism exhibited the most diverse relationships, linked to reduced trust and empathy toward out-group members, as well as heightened feelings of anxiety and disgust.

These living conditions, dysfunctional intergroup dynamics, institutional discrimination and challenges related to social integration form a basis for developing a framework to analyse the migration of the Roma population within European countries. Specific studies on the temporary migration of Roma from Romania and other European countries are limited (Toma and Fosztó, 2018; Corman and Croitoru, 2023; Piemontese and Maestri, 2023). Roma migration is often integrated into studies on the internal and international migration of the Romanian population (Toma et al., 2017; Delibas, 2023; Friberg, 2025). Existing research on temporary migration of Roma highlights various key aspects necessary for understanding the phenomenon, such as the influence of factors that lead to different models of temporary migration (Friberg et al., 2023; Tyldum and Friberg, 2022), the socio-economic conditions of migrants (Voiculescu, 2004), the role of social networks and support systems in facilitating temporary migration (Pantea, 2013; Anghel, 2024), as well as the implications generated by temporary migration (Corman and Croitoru, 2023), especially at the level of the country to which they migrate (Janko Spreizer, 2018; Hristova and Milenkova, 2021; Chatleska and Blazhevski, 2023; Jupîneant et al., 2024).

Although previous studies have explored this phenomenon, some have shown that migration factors vary from country to country (Knezevic Kruta, 2019; Sardelić, 2019). This exploratory study aims to complement existing data on the temporary migration of the Roma population from Romania, contributing to a better understanding of this complex process. In addition, we introduce the perspective of managers and representatives from places of origin regarding this process and its effects on their communities. As de Haas (2021) notes, there are no studies that identify the causes, consequences, and experiences of migration “from the perspective of the area of origin,” but rather studies that seek to understand migration mainly from the perspective of the destination country. Consequently, this paper aims to identify the driving factors that lead Roma people from Romania to temporary migration, the factors that motivate their return, and the effects of these movements on their communities. It does so by considering multiple perspectives—both those of Roma migrants and managers and representatives of public and socio-cultural institutions from the communities of origin. Therefore, the study addresses four research questions:

**Research question 1:** who are the Roma people who have experienced temporary migration for work?
**Research question 2:** what factors influence the temporary migration of Roma people?
**Research question 3:** what factors influence their return home?
**Research question 4:** what are the effects of the return process on their communities?

## 2 Theoretical background

### 2.1 Roma minority in Romania

According to data from the 2022 Population and Housing Census, in Romania, Roma people represent the second largest ethnic group, accounting for about 3% of the population (Population Housing Census, 2022). However, the number is likely higher, as the results depend on whether individuals As in most European countries, far from being a homogeneous group, Roma people are a population with a high degree of heterogeneity. The Roma are still identified today by certain specific aspects of their way of life, represented by: nomadism, poor living conditions—in carts, tents, or caravans, by specific trades and occupations—woodworking, metalworking, fortune-telling, singing, and customs related to marriage (Stoenescu, 2014). Fiddle music (“lăutăria”) is a traditional Roma occupation, one of the most famous traditional Roma crafts. This profession is passed on from father to son; it is practiced in groups, with the band, in taraf (Roma specific type of band), and more recently, at events such as weddings, parties, or fairs. Originally, fiddler did not mean you had to know the musical notes to practice it, because it was taught and learned “by ear”. The fiddle music is based on a large dose of improvisation, which is why some Roma fiddlers have now chosen jazz (Hertanu, 2020).

In addition to these aspects, language, clothing, and the way of organization from the perspective of laws are also very important for their identification. The duality of Roma in social relations refers to the fact that within Roma families, even if there are arguments and misunderstandings, they are honest and fair with each other, while they have a different attitude toward people who do not belong to the Roma ethnicity. The possible explanation is that the negative attitude is only a negative response to the discriminatory attitudes of non-Roma (Cherată, 1998).

In terms of education, the Roma prefer to educate their children themselves. They are taught to be responsible and to take care of their siblings. From an early age, girls are taught to take care of the household, and boys learn various trades (Grigore and Sarău, 2006; Hertanu, 2020). According to the European Parliament (2024), Roma children lag non-Roma children at all levels of education. Less than half (44%) of Roma children between the ages of 3 and 7 participate in early childhood education, compared with a 93% EU average for the same age group. While nine out of 10 Roma children aged between 7 and 15 are reported as attending school (88%), participation in education decreases significantly after compulsory schooling: only 27% of young Roma adults surveyed had completed their upper secondary education.

The number of Roma early school leavers is disproportionately high compared with the general EU population (71% compared to 9.7%). Moreover, school segregation remains a particular problem in Romania as in countries such as Bulgaria, Croatia, and Slovakia, despite the legal prohibition of this practice and case law of the European Court of Human Rights.

Fundamental elements within the Roma identity, prevalent across all Roma groups, encompass reverence for elders, and elder women, ritual cleanness, a dualistic understanding of the divine, attitudes toward death and the deceased, adherence to oaths, internal conflict resolution (particularly in familial matters) and generally the consensus-based community (Laederich, 2011). Their feeling of belonging to the community is strong, identifying themselves with the place where they live and having a clear sense of pride, as Luca (2023) underlined.

In the case of Romania, the Roma people are also in a situation of marginalization and social exclusion. The evaluation reports, drawn up both by representatives of civil society and by various international bodies and institutions, show that the Strategy of the Romanian Government for the inclusion of Romanian citizens belonging to the Roma minority for the period 2015–2020 has only partially achieved its objectives (Government of Romania, 2022). For example, the EU-MIDIS II report (FRA - European Union Agency for Fundamental Rights, 2017) shows that Romania was among the countries where 80% of Roma lived below the poverty risk threshold; the results of this survey also underline that one Roma in three lived in a house without running water; one in ten lived in a home without electricity; one Roma in four and one Roma child in three lived in a household where a family member went to bed hungry at least once in the last month. Housing deprivation requires at least one of the following dimensions: accommodation is too dark, has problems with humidity, has no shower/bathroom inside the dwelling, or has no (indoor) toilet. More than half of Roma households (52%) experience housing deprivation across all EU countries covered, with the highest rate in Romania (70%) (FRA - European Union Agency for Fundamental Rights, 2023, p. 52). The proportion of Roma living without tap water is higher in Romania (40%) than across EU countries surveyed (22%). In Romania, a lack of tap water is a problem for a substantial part of the general population (21%), resulting in a smaller gap between Roma and the general population. (FRA - European Union Agency for Fundamental Rights, 2023, p. 55)

In 2017, cases of discrimination and hate crimes continued to be reported, confirming the fact that stereotypes and prejudices against the Roma remain an important obstacle to their inclusion. Some studies show that existing prejudices against the Roma population have intensified during the COVID-19 pandemic, leading to special isolation measures for them during that period. Cretan and Light (2020) suggest that tensions between Roma communities, the police and other groups are not solely pandemic-related, but are also rooted in broader structural issues such as poverty and limited employment opportunities According to a survey conducted by FRA in 2021, the level of discrimination against the Roma population has hardly changed compared to 2016, in Romania as well as in other European countries included in the research. On average, one in four Roma respondents in 2021 (25%) felt discriminated against because of their ethnicity in the last 12 months in Europe, and the perceived discrimination rate in Romania is 20% (FRA - European Union Agency for Fundamental Rights, 2023, p. 21).

The strategy of the Government of Romania for the inclusion of Romanian citizens belonging to the Roma minority for the period 2022–2027 (Government of Romania, 2022), in the first section, evaluates the situation of Roma people from Romania in the last years. This official document underlined that in the field of education, there are still major discrepancies between Roma children and those of the majority population in terms of school participation, level of school performance, and school dropout, as well as regarding differential treatment, both in terms of the quality of the didactic act and discrimination and segregation. Among the main obstacles identified by the Council of the European Union in respecting the right to education, school segregation, early leaving of the education system and the low participation of Roma children in early education stand out in particular.

A possible cause that interfered with the implementation of the strategy for the inclusion of the Roma minority (related to the period 2015–2020) in Romania is that the action plans at the local level only reproduced the national measures, without adapting them either to the specific realities of the communities or to the level of available resources. The new national strategy aims to start from the specific realities of Roma communities and to be primarily focused on the development of national programs in the fields of education, employment, health, and housing. These areas remain the main pillars of intervention in disadvantaged communities, in the perspective of reducing the gaps with the majority population (Government of Romania, 2022).

Rostas (2019) suggested that the inefficacy of Roma policies arises from a confluence of historical maltreatment, discrimination, and poverty, resulting in the present challenges. He underscored the limited political influence of the Roma community and their minimal involvement in shaping relevant policies as fundamental reasons for policy inadequacies. Rostaş and Nodis (2022) noticed there are no comprehensive research projects on antigypsyism in Romania, about its manifestations, the mechanisms that produce and reproduce it, and its consequences for the Roma population. Consequently, there is a scarcity of public policies that effectively tackle the diverse issues faced by various Roma groups. Buhăescu-Ciucă and Ionită (2022) echoed this viewpoint, observing that policies often formally engage Roma in discussions without conducting thorough research into their specific circumstances.

The Roma population is confronted with a vulnerable situation in terms of employment, compared to the total population (Preoteasa et al., 2010; Preoteasa, 2011; Dănăcică, 2023). The situation of the Roma in the labor market remains problematic despite the active measures taken. A part of the Roma obtained a qualification at the workplace and benefited from the work experience, but the problem related to the reinsertion of the ethnic Roma population on the market remains open. Most of them, with a low educational capital for professional reconversion, cannot turn to other economic branches, or if they do, their absorption is reduced. In addition, Roma living in marginalized areas, including urban ghettos, negatively influence finding and maintaining jobs (Stănescu, 2010).

Occupational typologies of Romanian Roma have been developed after the data collected during research studies were analyzed. In a study conducted by Preoteasa et al. (2010), the identified occupational types are: Roma who “work on the black market”, Roma who “work wherever they can”, Roma with household activities, Roma with traditional activities, Roma with a job on the formal market, Roma with their own business, Roma who seasonal migrate abroad to work especially in agriculture. In statistical reports, Romanian Roma participation in the labor market and their level of professional qualification are below the national average (Cace et al., 2013; Horváth, 2017).

In their study, Corman and Sassu (2023) explored the question regarding where Roma works, and the answers were grouped as follows: abroad, in the country, or even in the locality of residence as day laborers. Migration especially seasonal migration is presented by Roma as a financial opportunity that allowed them to build a house and have a normal standard of living. Anghel (2019) underlined that seasonal employment opportunities abroad, alongside local occasional work and social benefits, serve as significant sources of income and contribute to enhancing housing conditions for disadvantaged Roma individuals and their families in Romania. Corman and Sassu (2023) found that the perspective of Roma people and the perspective of local social actors are quite different when presenting the jobs, the opportunities but also the risks arising from work activities, each perspective emphasizing something else. In the case of the Roma perspective, the reduced job opportunities, financial shortages, and difficulties, individual and community needs are accessed. In the case of the local social actors' perspective, training and qualification opportunities for Roma people, access to employment, and social benefits are offered, but also deviant Roma behavior is emphasized, as these imply risks in employment.

All these problems and challenges faced by the Roma population are generally for all, but they are particularly pronounced for Roma people in Romania. Firstly, their population is more numerous compared to other countries from Europe, and the state has not succeeded in developing adequate solutions to mitigate these issues. Compared to Roma from other countries, they are disproportionately affected by a lack of necessities, especially decent living conditions and access to tap water. Also, a significant proportion of Roma pupils do not complete their education, which leads to a lack of skills and qualifications necessary to access employment opportunities. These aspects can serve as significant factors influencing their migration, whether temporary or permanent, to other countries. Moreover, other countries, especially those with higher income levels, often have more effective strategies for Roma inclusion, providing better support to help them integrate into society. There is an ongoing focus on developing new social policies for their integration, driven by several reasons, including economic considerations. Roma individuals could significantly contribute to the workforce, but they face challenges such as a low rate of school completion, low employment rates, and a high tendency to migrate, either temporarily or permanently.

### 2.2 International migration of the Roma population in Europe

Migration refers to the movement of people from one place to another, intending to settle temporarily or permanently in the new location. United Nations recommendations for this concept is: “In the global context, movement of a person either across an international border (international migration), or within a state (internal migration) for more than 1 year irrespective of the causes, voluntary or involuntary, and the means, regular or irregular, used to migrate” (European Commission, n.d.). Its measurement depends entirely upon how it is defined in time and across space. Kosiński and Prothero (2023/1975) underlined that migration is much more related to a permanent change of residence, compared to the term mobility. An operational definition of migration requires that both temporal and locational criteria be more specifically defined.

Migration is commonly perceived as a shift from an individual's typical place of residence, yet it seldom entails a singular, straightforward journey. Individuals often move back and forth, engaging in short-term spatial transitions as well as longer-term stays. Numerous seasonal migrants fall under the category of circular migrants, characterized by recurrent migration cycles between an origin and destination, encompassing multiple instances of migration and return. They often bring back improved skills and fresh perspectives to their home community and can help develop networks with destination countries (Hugo, 2013; Lam and Rui, 2023). Seasonal and circular migrants can return to the host country, including the same workplace, to perform the same work as in previous years. In such instances, employers can minimize the need for extensive training and supervision of the workers.

Studies on the migration of Roma populations in European countries have explored various aspects of both permanent and temporary migration (e.g., Piemontese and Maestri, 2023; Corman and Croitoru, 2023). These studies often investigate the socio-economic conditions, cultural factors, and policy implications surrounding Roma migration. For example, a study by Vlase and Voicu (2014) shows that Roma people can be active agents in shaping their living conditions and their interactions with public institutions, with migration serving as a deliberate strategy to improve their economic and social circumstances. Research on permanent migration among Roma populations often examines factors such as discrimination, marginalization, and lack of socio-economic opportunities in their countries of origin. Permanent migration may be driven by seeking better living conditions, access to education, healthcare, and employment opportunities in destination countries (Stewart, 2013).

Temporary migration of Roma populations may involve seasonal work, nomadic lifestyles, or short-term mobility for economic reasons. Temporary migration patterns are influenced by factors such as the availability of informal labor markets, social networks, and historical traditions. For instance, a recent study analyzed the fact that Scandinavia has emerged as a new destination for Romanian Roma engaging in circular migration for begging and street work (Friberg et al., 2023). Tyldum and Friberg (2022) describe how Roma migrants to Scandinavia organize their travel through tight-knit family networks that provide social support and information, allowing people to engage in circular migration despite having limited formal and economic resources at their disposal.

Research on the temporary migration of Roma populations has focused on several key aspects, including the drivers of migration, patterns of movement, and the socio-economic conditions of migrants both in their home country and destination countries. As an example, research conducted by Voiculescu (2004) emphasized that the level of deprivation and scarcity of resources, such as agricultural land and formal employment opportunities, coupled with proficient knowledge of the Hungarian language, have prompted Roma communities in Transylvania, particularly in Harghita County, to engage in temporary migration to Hungary. As a result of migration-associated practices, their economic and social dynamics have started to evolve, although they have not replaced their local means of sustenance. The economic drivers of temporary migration among Romanian Roma, include factors such as poverty, unemployment, and lack of opportunities in their home communities. Additionally, studies have examined the livelihood strategies employed by migrants to sustain themselves during their temporary stays in destination areas. For example, Harrison et al. (2022) underlined that the Roma population, having endured a prolonged and symbiotic relationship with precarity, exacerbated by centuries of persecution, provides valuable perspectives into the first-hand realities of precarious workers.

Corman and Croitoru (2023) identified the hidden costs of seasonal migration and discussed them at three levels of analysis: individual, familial, and community. Even though migration is the most significant factor of social change in the studied Roma communities, and its effects are multifaceted, it also implies significant negative costs of migration in terms of health, education, employability, family, and community life. In the medium and long term, these effects decrease the positive aspects linked to the material gains from migration, making these Roma communities more vulnerable and dependent.

The role of social networks and support systems in facilitating temporary migration has also been investigated (Anghel, 2024). Pantea (2013) underlined that even amid severe poverty, social networks wield significant influence over migration decisions. She underscores that migration patterns are often specific to communities and influenced by a locally shared culture (ethos) on migration. These networks often play a crucial role in providing information, resources, and assistance to migrants during their journeys and stays in destination countries. Toma and Fosztó (2018) discovered that social networks play a dual role: they enable migration while also aiding in the redefinition of social categories within the home community. They distinguished two primary patterns of network formation: in some areas, network connections traverse ethnic lines, fostering robust interactions and communication among diverse segments of the local population; conversely, in other instances, network connections are predominantly confined within ethnic groups, with limited ties extending across ethnic boundaries. Although there are certain differences between the way of life and the behavior of the Roma depending on the area they come from, and the communities they come from, a common aspect of them is represented by the fact that their migration process is usually carried out through social networks, through family members who are already in the country of destination. Roma are assisted by family and migrate with the help of social networks, claiming that they also return home mainly to be close to family (Bîrsan and Hirian, 2011).

Studies often explore the challenges of social exclusion and integration faced by migrating Roma populations (Janko Spreizer, 2018; Hristova and Milenkova, 2021; Chatleska and Blazhevski, 2023). These include issues related to access to housing, education, healthcare, and discrimination in host countries (FRA - European Union Agency for Fundamental Rights, 2017). To cope with such challenges, Roma migrants adopt various strategies. Some studies (Jupîneant et al., 2024) highlight that Roma migrant women play a crucial role in providing support for Roma migrants, particularly by taking responsibility for child-rearing and the preservation of Roma cultural identity, especially during periods of crisis such as the COVID-19 pandemic. The FRA - European Union Agency for Fundamental Rights (2017) survey provides insights into the living conditions, discrimination, and social exclusion experienced by Roma communities across various European countries, highlighting the significant challenges they encounter in the process of social integration.

Researchers and policymakers have analyzed the effectiveness of policies designed to address the needs of migrating Roma populations, including integration programs, anti-discrimination measures, and efforts to improve socio-economic opportunities (European Roma Grassroots Organisations Network, 2020). This document promotes Roma integration, including initiatives to address migration-related issues. These studies (i.e., Brüggemann and Friedman, 2017; Iusmen, 2018) contribute to a better understanding of the complex dynamics of Roma migration in European countries and inform policy discussions to promote social inclusion and address the challenges faced by Roma communities.

### 2.3 Push-pull theory

Due to the complexity of migration explanations, there is no single overarching paradigm that can fully justify migration or generalize and systematize all empirical research findings on the topic (de Haas, 2021).

Many theories from the economical or sociological approach (O'Reilly, 2022; Massey et al., 1993; Becker, 1962; Borjas, 1994; West, 2011; Massey et al., 2005) explained the migration process, but we considered that the most appropriate theoretical framework for our study was the push-pull theory. Initially, we considered that the theory of economic origins is the basis for explaining the migration process. These suggest that economic factors are the primary drivers of migration. However, relevant studies (Carling and Collins, 2017) suggest that the determinants of migration need to be reconsidered in relation to migrants' subjectivities. This perspective is echoed by Black et al. (2011), who identify five drivers of migration: economic, political, demographic, social and environmental. Building on this framework, Van Hear et al. (2017) further propose a differentiation between predisposing, proximate, precipitating and mediating drivers. Nevertheless, a central challenge remains: determining which factors are most significant, under what conditions they gain importance, how they interact, and how these interactions evolve.

Based on data about the Roma population is known that a significant proportion of individuals are unemployed, and they appeal to migration to improve their income, but the process of their return after a while makes us think that other factors are involved in this process of Roma migration. In the push-pull theory, Everett Lee proposed that migration is influenced by push factors that drive people away from their place of origin and pull factors that attract them to a new destination. Push factors may include poverty, lack of job opportunities, political unrest, or environmental disasters, while pull factors may include better economic prospects, political stability, or social networks in the destination area (Lee, 1966). This author underlined that people react differently to push-pull factors, based on differences in age, gender, and social class, differences that influence them, as well as their ability to overcome the obstacles they encounter.

International migration can be driven by various factors such as economic opportunities, political instability, social reasons, or environmental conditions. Migrants are in search of better opportunities related to education, employment, and living standards, among other factors. However, as de Haas (2021) also identified in his studies, migration is a counterintuitive phenomenon, more specific to developing countries, where marginal incomes increase, education and health systems improve, as well as infrastructure. In other words, development initially leads to more pronounced migration. This shows us that migration is chosen not only to find better and better-paid jobs, where education and health systems are better, but there are other, deeper motivations for leaving. Understanding migration involves exploring the motivations behind people's movements and the impacts on both the origin and destination areas.

We considered it important to understand Roma migration and their return home, the causes and consequences generated, considering that, as de Haas et al. (2020) also specify, these phenomena can generate transformations at several levels, with profound implications on the social structure but also on the geographical distribution of the population. We chose the push-pull theory in the context in which it best theorizes, in our opinion, both the factors of migration and those of return home. de Haas (2021) considers that one of the factors contributing to the lack of progress in the general understanding of migration is the “receiving country bias” and the fact that studies ignore research on the causes, consequences and experiences of migration “from an origin-area perspective”. We believe that by identifying the pull-push factors of the external migration of Roma from Romania, we can complete the picture of the push-pull factors described in Lee's (1966) theory and we can identify the deeper causes of migration, their consequences and the experiences of migration from the perspective of migrants, but also of those who remain in the “area of origin” (local authorities).

## 3 Materials and methods

### 3.1 Data collection method

For this study, we used a convergent mixed-methods design (Creswell and Clark, 2017). The convergent design involves collecting quantitative and qualitative data simultaneously, and both strands have equal emphasis. We merged quantitative and qualitative data to better understand the complexity of the temporary migration of the Roma people. The quantitative part of the study aimed to explore the perspectives of the Roma population regarding their reasons for migration and return. During this stage, we collected data through a non-standardized questionnaire from 796 Roma individuals who had experienced international temporary migration, but currently live in Olt County, Romania. Data were collected in a non-probabilistic way, appealing to the support of their informal leaders and cultural mediators in those areas.

The data collection process took place between July and December 2023. It was challenging due to a high non-response rate, primarily caused by mistrust or fear of revealing personal information. This led to the process extending over such a long period. To overcome these difficulties, we also relied on the support of staff from local institutions who work directly with the target group. Only individuals who agreed to complete the questionnaire were included in the final database. In this database, 95% of the respondents answered all the questions. One measure taken to prevent participants from abandoning the questionnaire was to design a shorter version with simple, easy-to-understand questions. Some participants were assisted in completing the questionnaire due to difficulties with reading or writing. On average, completing the questionnaire took around 15 min.

The qualitative part aimed to capture the perspective of the social actors who are leaders of various institutions in the same areas. This included examining both the reasons behind Roma migration and the effects of their return on their communities. We collected qualitative data through semi-structured interviews with fifteen managers and representatives of public and socio-cultural institutions from the same region during the period October to December 2023. As in the previous phase, the data collection period was extended due to the busy schedules of these stakeholders.

One limitation of the research design was related to the length and format of the questionnaire, which did not allow for in-depth insights into the perspectives of the Roma population. Based on previous studies reporting high non-response rates, we decided to use a short questionnaire with closed-ended questions to ensure completion. However, this choice limited our ability to explore their views regarding context and experiences of migration in depth. This limitation was partially addressed through qualitative data from the representative, which helped provide a more comprehensive understanding of the migration process. Due to their limited availability, we developed a short interview guide to ensure their willingness to participate. The average duration of these interviews was about 40 min.

The results obtained from these stakeholders largely overlapped with those from the Roma population but also revealed additional socio-cultural factors influencing both migration decisions and the decision to return. The findings supported our initial expectations by identifying economic factors as the primary driver, but stakeholders also highlighted discrimination as a key factor influencing the decision to return. Additionally, our study succeeded in identifying specific indicators within both the economic and social factors categories (see Tables A1 and B1).

Furthermore, the results provided insights into the broader context in which participants live, which likely influences their migration decisions. The qualitative data also highlighted particular aspects, especially socio-cultural ones, offering a more comprehensive picture of the migration process. Moreover, the perspectives of stakeholders offered valuable insights into how the return process affects both individuals and communities, providing useful input for the development of future policies and integration efforts. The research received the approval of the Ethics Commission in Social Research from Transilvania University of Brasov, Romania. The participants in the study received information at the beginning of the questionnaire/interview about the purpose of the survey and the informed consent procedure.

### 3.2 The research instruments

The questionnaire applied to the Roma population included items corresponding to the first three research questions. To discover the profile of Roma people who had worked abroad at least once, questions regarding socio-demographic characteristics were included (see Table 1).

**Table 1:** Sociodemographic characteristics of respondents.

**Variables**	**Category**	**Count**	**Percent**
Gender	Male	406	51%
	Female	390	49%
Age	18–30	202	25.4%
	30–40	241	30.3%
	40–50	199	25.0%
	50–60	113	14.2%
	over 60	41	5.2%
Residential environment	Rural	164	20.6%
	Urban	632	79.4%
Years of study	Primary school (grades 1–4)	195	24.5%
	Middle school (grades 5–8)	276	34.7%
	High school (grades 9–12)	189	23.7%
	I didn't go to school	136	17.1%
The place where they live (q8)	In a block of flats	104	13.1%
	At house	645	81.0%
	In another space	32	4.0%
	Rent	2	0.3%
	Tent	2	0.3%
	Social housing	2	0.3%
	Don't answer	9	1.1%
The job (q10)	Do you have a stable job	82	10.3%
	Do you not have a stable job	478	60.1%
	Are you looking for a job	233	29.3%
	Don't answer	3	0.4%
The frequency with which they go abroad (q19)	Once a year	259	32.5%
	Twice a year	305	38.3%
	Three times a year	91	11.4%
	More than three times a year	120	15.1%
	Don't answer	21	2.6%
The country where they go to work (q17)	Spain	223	28%
	Italy	180	22.6%
	United Kingdom	182	22.9%
	German	125	15.7%
	France	11	1.4%
	Sweden	8	1%
	Other countries	41	2.5%
	Don't answer	26	3.3

For the second and third research questions, items were included to directly measure the factors that influence Roma people to go abroad for work or to return to Romania. These items included questions such as the main reasons for leaving Romania (q16), the main reason they refuse a job (q10), the aspects they like most about the country they chose to go to (q18), the reasons why they return to Romania after a certain period (q21), their living conditions in the country they migrate to (q23), how they feel that are treated in other countries compared to Romania (q24), and how citizens react when they ask for help abroad compared to people in Romania (q26).

We also measured indirect factors, which are general conditions that lead to migration but are not necessarily the primary reasons. These included questions such as how satisfied they are with their life (q15), their living standard (q2), their social assistance received from the Romanian state (q3), the medical services they received (q14), the biggest difficulty they face in Romania (q29), their relation with neighbors (q9), the reasons they refuse a job (q12), their opinion about whether the Romanian state is taking actions to facilitate their access to education (q4), whether they feel judged in Romania because of their ethnicity (q31), and whether they feel their rights are respected in Romania (q28), the people with whom Roma individuals go abroad (q21).

To capture the perspective of managers and representatives of public and socio-cultural institutions, we used interviews as a method. The interview guide was structured around topics related to the external migration of Roma and the impact of their return home. These topics included the reasons why Roma left Romania, the reasons why they returned, and the effects of their return migration on Romanian society. Both instruments collected much more information on the Roma population but in this article are presented only those that refer to the migration process.

### 3.3 Participants

These data that describe the participants of our study also respond to the first research question. The participants were from one area of Romania, the named Olt region. Participants were in equal measure male (51%) and female (49%) from all categories from age: 25.4% are aged between 18 and 30 years, 30.3% are between 30 and 40 years, 25% are between 40 and 50 years, 14.2% are between 50 and 60 years, and 5.2% are over 60 years old. Regarding the area of residence, most respondents come from urban areas (79.4%), while only 20.6% are from rural areas. Concerning the education level of the respondents, 34.7% have middle school as their highest level of education, a relatively high percentage of 24.5% have completed only primary school, 23.7% have graduated from high school, and 17% have no formal education. The majority of them lived “at the house” (81%). Only 10.3% have a stable job, and most of them go abroad once a year (32.5%0, and twice a year (38,3%) (Table 1). Roma people prefer as the place of migration countries such as Spain (29%), Italy (23.4%), the United Kingdom (23.6%), Germany (16.2%), and France (1.4%) or Swedish (1%) based on their networking and previous experience of migration. According to data from the 2022 Population and Housing Census for Romania, the migratory rate is the same in preferences as the rest of the other migrants. The existing networking helped them to find a job in these countries but also the information they can access from others with migratory experience.

### 3.4 Data analysis

The analyses of the two types of data were done separately using specific procedures for analysis. There are multiple ways to present data based on mixed methods (Creswell and Clark, 2017; Wittink et al., 2006), but in this study, we analyze separately those two kinds of data and we combine the results in the part of the Discussion section. Quantitative data are presented in Table 1 and Supplementary material (Table A1) in a descriptive manner framing data in a push-pull framework.

We compare quantitative data with qualitative data (for research questions 2, and 3) to confirm and to complete data from the perspective of the Roma population with from managers and representatives of public and socio-cultural institutions and to see in what ways the two sets of results converge or diverge. For qualitative data, we used a directed (deductive) approach to qualitative content analysis, as we started from a pre-existing theoretical framework (Assarroudi et al., 2018; Hsieh and Shannon, 2005). The qualitative data were analyzed and grouped into categories predefined by the Push-Pull Theory. Four categories were defined: push factors from Romania, pull factors to other countries, push factors from other countries (for returning), and pull factors to Romania (for returning). The first two categories explain the reasons why Roma people choose to migrate abroad, while the last two categories explain the reasons for their return home. For each factor, we further defined two subcategories: economic factors and socio-cultural factors. Qualitative data for research questions 2 and 3 are presented in the Supplementary material (Table B1).

For research question 3, we used also a deductive approach to qualitative content analysis, and we grouped the information into two categories: positive effects and negative effects of the return process on the communities (See Supplementary material, Table C1).

To ensure the validity of the results, the qualitative data analysis was conducted separately by two investigators. Subsequently, they discussed their findings and reached an agreement regarding the final categories (Bengtsson, 2016). Additionally, to ensure the quality of the analysis, the data are presented in the form of a categorization matrix (Assarroudi et al., 2018).

## 4 Results

### 4.1 What factors influence the temporary migration of Roma people?

We start our discussion with indirect factors (see Table A1) that create a negative context that pushes individuals to consider migration as a long-term solution. There are two categories of factors that contribute to this context economic factors and socio-cultural factors. The economic factors refer to general poverty, unemployment, no specific job opportunity, or income disparity that makes migration appealing as a potential solution. Therefore, 47.2% of them consider that consider their standard of living to be bad. 57.1% are very dissatisfied or dissatisfied with the social assistance provided by the Romanian state and 30.2% consider that the medical services provided are very bad. 62.7% consider the lack of money to be the biggest difficulty they face in Romania, 20.2% identify finding a job as their main difficulty, 6.2% see buying a home as their biggest difficulty, and 4.1% consider the lack of access to water or heat as their primary concern.

39.4% are very dissatisfied with their lives, 48.4% consider that their rights are not respected, 37.9% believe that the state takes no action to facilitate access to education for Roma people, and 44.2% report feeling disadvantaged compared to other ethnic groups.

Even though 49.3% consider that they are largely judged because of their ethnicity, only 4.5% have bad and very bad relations with their neighbors, and 5.3% consider the lack of respect from others to be the biggest difficulty they face in Romania.

Overall, these findings suggest that Roma people live in a context with significant negative impacts that might act as sources of migration. While they express dissatisfaction with discrimination and the attitudes of others, they do not explicitly identify these factors as primary reasons for migration.

The direct factors that have an immediate impact on their decision (see Table A1), based on what they declared as the main reasons, are primarily economic. The majority lack stable jobs (89.4%) and are motivated by the desire to find a job (10.2%), seek a better life (67%), get out of poverty (13.3%), or earn money to build a house (2.5%). However, even when they can work in Romania, many refuse to work, due to low wages (70.3%) or the difficulty of the work (11.4%). This highlights the critical role of economic factors in driving their migration decisions.

The pull factors that attract them to other countries are principally economic. Therefore, 72.4% declare that they migrate to specific countries because of the opportunity to make money. Another factor is the place where they can live, a place where they don't pay: 14.5% migrate they live in relatives' homes, and 3.7% migrate they live in houses provided by their employers. The socio-cultural context also influences their decision to return to those countries. A significant proportion (18.3%) cite the way people treat them, while 4.3% cite the climate. Furthermore, 71.3% feel they feel treated better in other countries, 76.5% report facing fewer difficulties abroad compared to Romania, and 67.4% believe that foreigners are more willing to help them than Romanians. These factors emphasize the importance of social acceptance and better living conditions in shaping their migration choices.

The data collected from interviews with managers and representatives of socio-cultural institutions that interact with the Roma population confirm the economic push factors for external migration, previously detailed by the Roma respondents, but also come with additions related to pull factors that influence the external migration of the Roma (see Table B1). The pull factors to other countries are based, first of all, on the Roma belief that they will earn more money, will obtain financially satisfying jobs, and will have a better life abroad and a higher salary level than in Romania. But above all, a great impact is the recommendation of members of the social network who are already in the host country or have been there previously to work, and who provide them with this kind of information. In other words, the existence in the social network of an acquaintance who works abroad is a factor that has an important role.

In addition, the managers and representatives mention socio-cultural push factors: the discrimination, the lack of social integration programs, the nomadic tradition of the Roma – “*the desire for the new, it is known that they were and still are nomads*”, “*because they were originally nomads and they want to know new societies*”. The favorite destination countries for Roma are predominantly Latin, phonetically and lexically similar to Romanian, with languages that are easy to learn – “*because of linguistic similarities*”. These countries are also similar culturally and temperamentally with Romanian people - “*there are countries of Latin origin and the population has a more developed philanthropic spirit than the Nordic peoples*”-, they have immigrant-friendly social policies – “*there are countries that have permissive legislation toward migrants*” -, lower level of discrimination – “*overcoming stereotypes regarding ethnicity by the inhabitants of Spain, Italy*”. Other socio-cultural pull factors are cultural diversity and higher opportunities for vertical social mobility – “*for new development opportunities that they don't have in Romania*”-. Another pull factor mentioned by managers and representatives is the friendly climate in countries such as Spain and Italy. Roma also mentioned this aspect, but a small segment of 4.5%.

The responses of Roma who migrate focus mainly on mentioning economic factors, compared to those of managers and representatives of cultural institutions who rather mention socio-cultural factors of migration.

Although we could not identify it as a pull factor in the initial migration decision, we found that subsequent migration can be influenced by satisfaction with the host country. This variable includes, on the one hand, higher financial earnings for the work performed and, on the other hand, the attitude of people in the host country toward Roma people. The perception of Roma regarding the attitude of the majority population toward them is significantly different in the context of the destination country and that of the country of origin. Most Roma believe that they are better treated and respected in the host country than in Romania, although this was not identified as an initial reason why Roma decided to migrate. We note that, although discrimination is not a push factor for initial migration, compared to the positive and non-discriminatory attitude of people in the host country, we can, however, consider it a pull factor for secondary, subsequent migration, after a return.

Another pull factor that could influence the decision to migrate, later after a return, could be the way of helping and attitude of citizens in the host country. Most respondents believe that citizens of the host country are more willing to help them than Romanians.

### 4.2 What factors influence their return home?

If, for the reasons behind external migration, the Roma primarily identified economic factors as their motivation to leave, we observe that for returning home, they identified sociocultural factors as the main reason (see Table A1). For the Roma, the main pull factor for returning home is family. 74.1% return to be with their families, 12.4% because they have a house in Romania and feel at home, and 2.7% because they have friends in the country. Even though they go abroad with their spouse or other relatives (41,9%), and 21.5% go abroad with friends, they do not feel at home except in Romania, where their extended family is. For them, it is important to be together with their communities, special their extended family or their network of friends. Only a segment of 33.3% migrate alone, and for them, the feeling of being alone is strong.

A second reason for their return is that they feel treated worse abroad (10.5%), 9.1% face more difficulties there than in Romania, and 13.7% perceive foreigners as less willing to help them compared to Romanians.

From the analysis of quantitative data, the economic push factor that generates the return of Roma to Romania is the one referring to the difference between the expectations with which the Roma migrated and what they experienced in the host country. The perception that the Roma have about Romania can be summarized in a typology that includes two categories: two Romania(s)–family/home Romania and gloomy Romania (poverty, corruption, disaster). “Family Romania” is associated with family relationships, birthplace, and feelings of belonging to a community, while “gloomy Romania” is associated with a low standard of living, daily material and financial difficulties, and a socio-economic context unsuitable for development. Thus, the research subjects have an ambivalent relationship toward migration: on the one hand, they migrate to the West with the thought of a better life for themselves and their family, but they return home, missing their family.

Qualitative data (see Table B1) complete the reasons for the return of Roma to Romania. From the interviews with managers and representatives of cultural institutions, we find that the push factors for returning are the lack of integration into the labor market in the host country: “*the difficulties faced by Roma in terms of inclusion in the labor market*”, the lack of social integration in the host country, but also the avoidance of punishments: “*they often break the law of the respective country and return home to escape from the rigors of the law*”.

As pull factors reported by the managers and representatives of cultural institutions, in addition to the missing of the family and birthplaces – “*the family and the environment from where they come are important factors for returning of Roma to their country of origin*” -, factors also mentioned by Roma who are returning to Romania, the subjects of the qualitative research recall the fact that in Romania, Roma can authentically express and manifest their traditions and customs – “*the Romanian national space is attractive and hospitable and Roma traditions are anchored in customs and habits that can only be expressed in Romania*”, “*they are deeply connected to everything that culture and tradition mean*”. In addition, the data shows that managers and representatives perceive Romanian legislation as more relaxed – “*Romanian legislation is not as demanding as that of the countries where they migrate*”. Another pull factor refers to investing in Romania, the resources accumulated in the country of migration for a better life but also to obtain social validation from the community (the desire to boast and assert oneself in front of those who remained home).

### 4.3 What are the effects of the return process on their communities?

This question could only be analyzed in the qualitative research, from interviews with the managers and representatives of cultural institutions, who interact with Roma people. Among the themes of the interview guide, we also wanted to identify the effects of the return of Roma to Romania and the dimensions that these can generate, at the community and societal level. Thus, from the qualitative data, it emerges that the phenomenon of Roma return migration can have both positive and negative effects on the community (see Table C1).

The positive effects of Roma returning home are reported less than the negative effects. The managers and representatives of cultural institutions in the country highlight the positive effects of Roma returning home by pointing out that Roma returnees could contribute to the growth of the economy because “*they bring money into the country that will be spent here*”, “*they have invested, opened businesses and provided jobs to people from their community*” and contribute to “*raising the standard of living*”. At the same time, the cultural exchange the Roma have experienced in the host country has positive effects on them, with an impact on the community: “*the emergence of customs specific to the countries from which they returned*”, “*moving from begging to the villa*”. The migration experience means, for some Roma, the awareness of the role of education, with an increase in the rate of enrolment/re-enrolment in the education system, as well as the fact that “*if they've been to school when they come back, they equalize their studies from abroad*”.

As negative effects, managers and representatives of cultural institutions highlight the increase in the crime rate due to the inappropriate behavior of some Roma – “*if only Roma who created social problems return, then the effects are negative*”, “*the number of thefts increases*”-; the increase in unemployment due to the lack of education and qualifications required on the labor market – “*the emergence of an increasing number of unemployed*', “*constant obstacles in accessing the labor market, due to poor training and unequal access to quality education*”, “*the labor market cannot absorb them*” but also difficulties regarding the education of children who migrated with their parents “*the general difficulty of children's readjustment to school*”.

A problem reported by interviewees consists in the fact that Roma migrants - through their actions and lifestyle (e.g., “*outside they deal with begging and stealing*”)- leave behind them in the host countries a generalized negative image about an entire nation – “*Roma ethnicity is confused with Romanian nationality*” - and generates “*a decrease in foreigners' trust in Romanian citizens*”.

## 5 Discussions and conclusions

Our study presents a current phenomenon that Romanian society is facing, that of temporary external migration of Roma, with a focus on the push-pull factors involved in migration decisions. The results of this study highlight the presence of explanations of the push-pull theory in the process of external migration of Roma but also bring new perspectives for understanding and approaching this phenomenon. Our results underline that the decision to migrate is driven by push-pull factors that differ from the push-pull factors of returning to the country; this difference is also supported by other studies (Van Hear et al., 2017). The present study also highlights the various perspectives of Roma and managers and representatives of cultural institutions, regarding the driving factors that generate Roma's external migration: Roma people tend to identify mainly the economic factors that lead to migration, while managers and representatives also talk about the socio-cultural factors involved in the Roma people's decision to migrate. Thus, the perspectives of Roma and managers and representatives of cultural institutions are different regarding the push factors that generate external migration of Roma, with Roma identifying only economic factors that lead to migration, while managers and representatives also talk about socio-cultural factors involved in the decision to migrate Roma.

Regarding pull factors, we can observe that the results from the present study revealed new pull factors of migration to certain countries. The push-pull theory can be updated and completed with new dimensions, such as the friendly climate and the easier learning of the language of the host country. For Roma migrants, who intend to return to their community of origin, family is the main factor for returning to Romania. Another factor is represented by the disappointment of the migration experience, due to the lack of information and education of the Roma. The decision to migrate as well as that to return home can be explained by the lack of realistic information of the Roma about what being abroad means, there being huge differences between expectations and reality.

Compared to the assumptions of the push-pull theory (Lee, 1966), supplemented by the migration factors of Van Hear et al. (2017) in our findings, we encounter only two categories of push factors for the external migration of Roma: poverty and lack of job opportunities, as predisposing and precipitating factors for migration. Among the pull factors specified by the previously mentioned theory, we explicitly found in the collected data only the category of better economic prospects, as a proximate factor for migration. Another pull factor for external migration is represented by social networks, as a mediating factor. This is found in our study only in the form of recommendations made by network members regarding the country of migration destination, not necessarily as a well-built migration network.

At the same time, our findings bring new perspectives for understanding and ranking these factors, depending on the perspectives from which we look at the phenomenon. Thus, our findings suggest that the factors that generated the decision to migrate externally are different from the perspective of Roma and that of cultural institution managers and representatives, as other studies suggest (Corman and Sassu, 2023). The responses of Roma who migrate focus mainly on mentioning economic factors, compared to those of cultural institution managers and representatives who rather mention socio-cultural migration factors.

One of the explanations for the economic factors mentioned by Roma is the fact that their access to the labor market is still reduced (FRA - European Union Agency for Fundamental Rights, 2017), and working abroad is identified as a source of income to a large extent for Roma. The economic push factors identified in our study by Roma are: poverty, low-paid jobs, unstable jobs, low living standards, but also the lack of jobs. These are significantly correlated with the low level of education of the respondents in this study. The same data show us that Roma people with high school education believe that life in Romania is good and have no intention of migrating. Thus, we see that the data from our research confirms what other studies say: the higher a person's educational level, the higher their chances of employment and the better paid and more stable their job is (European Commission/EACEA/Eurydice/Cedefop, 2014; Deloitte and NHRD, 2021).

Similar studies conducted in other countries on temporary migration of Roma have shown that migration factors differ from country to country. For example, Sardelić (2019) shows that in Slovenia, the main push factors for which Roma migrate temporarily to Austria are the lack of jobs and the lack of real opportunities to progress in their careers, and among the pull factors of migration we find the possibility of earning higher salaries, factors that we also find in our research. However, Roma in Slovenia identify discrimination as a push factor of migration, which Roma in Romania do not specify, it being mentioned as a push factor only by managers and representatives of cultural institutes. Another research conducted on Roma migration from Serbia (Knezevic Kruta, 2019) shows that the main factor in their migration is political, with them seeking asylum in the destination countries, a factor that we did not find mentioned in our research, neither by Roma migrants nor by managers and representatives of cultural institutions.

One of the pull factors for Roma external migration identified in our study is that Roma is more likely to migrate to destinations recommended by members of their social networks. As shown in other research (Bîrsan and Hirian, 2011), Roma are more likely to migrate to countries recommended by people in their social networks, preferring destinations where other family or community members are already present or destinations that other members of the social network have experienced. This idea is found in the theory of migrant networks, which represents “sets of interpersonal ties that link migrants, former migrants, and non-migrants in origin and destination areas through the bonds of kinship, friendship, and shared community origin” Massey et al., 1993, p. 396). Transnationalism theory also emphasizes the importance of Roma migrants maintaining connections with their country of origin while carrying out social and economic activities in host countries. Through transnational networks, Roma communities support each other to preserve their culture and capitalize on economic opportunities, which contributes to their resilience to the challenges of migration (Ullah et al., 2024). These types of connections can also be identified in the case of Roma who migrate temporarily for work.

Among the socio-cultural push-pull factors highlighted by managers and representatives of cultural institutions for the external migration of Roma, discrimination and the nomadic lifestyle specific to the Roma ethnic group are worth mentioning. These factors are also found in other researches such as Friberg et al. (2023) and Tyldum and Friberg (2022).

We can also observe a correlation between the Roma's preferences for migration to certain countries, especially Latin countries, which facilitate their easier learning of the host country's language. Pull factors that we did not find mentioned in other studies were the possibility of learning the host country's language more easily and its climate, as well. This also explains the preference of the Roma in our study for migration to countries such as Spain and Italy, countries similar in linguistic terms to Romanian, all of which are Latin countries. These countries also have a more friendly climate than other countries to which Roma migrate temporarily, to a lesser extent, such as England and Germany.

Along with the push-pull factors of external migration of Roma, our study also aimed to identify the factors of the return of Roma to Romania, factors that we chose to analyze using the same conceptual framework of the push-pull theory. It is noteworthy that both the decision to migrate and the decision to return home can be explained by the lack of realistic information of Roma about what meaning life abroad, but also by their low level of education. Dragan et al. (2025) show that a strong predictor of adaptation to a new country is the level of education. Thus, their study shows that women with higher education manage to have the highest rates of adaptation to the new destination. In our study, we found that Roma go abroad with high expectations, convinced of the better life that the inhabitants of the host country have and that they will also achieve, and when they get there they realize that they face the same barriers they have in Romania: inaccessible jobs due to low education and jobs paid according to the education they have.

In addition, our data shows that Roma migration comes with a non-monetary cost that they perceive after arriving in the destination country: the family and birthplace missing, which is what drives them to return home. This is the main reason why Roma return home, to be close to family, as other studies show (Bîrsan and Hirian, 2011). In another study regarding the external migration of Polish Roma, migration carried with itself different costs, which can be observed in the forms of managing intragroup solidarity and cohesion as well as tensions related to, for example, the extension of social control beyond the border and attempts to preserve the norms and traditions (Fiałkowska et al., 2024).

Migration is an experience that brings with it both positive and negative effects, both on those who migrate, on the host country, and on the community in which they live. Differentiated migration strategies and practices of Roma people create very different return migration models both in terms of the profile of migrants and in terms of the financial and knowledge potential accumulated by migrants, as Anghel (2019) also underlined. The effects of Roma return migration are complex and varied, being influenced by factors such as the economic and social context of the country of origin, experiences abroad, and measures adopted by the authorities to support reintegration. Anghel (2019) also identified three major categories of returnees: migrants who are involved in temporary mobility practices and who return constantly (or for longer periods of time), migrants who return and find work locally and, finally, migrants who return and become entrepreneurs, sometimes successful.

From our research data, we can analyse the effects of Roma returning home on several dimensions.

### 5.1 Economic effects

As other studies show (Corman and Sassu, 2023), one of the most visible positive effects identified in our study is related to financial gains in the host country. Returning to Roma sometimes brings savings accumulated abroad or work experience, which can contribute to improving the economic situation of their families and communities, but also to building a house and having a decent standard of living. Anghel (2019) discovered that the majority of those who return are those involved in temporary migration, not necessarily those who have lived abroad for a long time. In addition, it has been observed that those who are more successful in returning to their country of origin are those who have spent more time abroad. At the same time, in the absence of economic opportunities in their countries of origin, return can lead to unemployment and increased poverty, especially in marginalized communities. Beyond the positive economic effect that migration plays, it is relevant to note that through migration and return, economic inequalities most often do not disappear, but are reproduced, and sometimes modified.

### 5.2 Social and cultural effects

From a social point of view, after the experience of living in other countries, Roma may encounter difficulties in reintegrating into their communities of origin, especially if the same precarious conditions persist. One of the positive roles that cultural exchange implies, as shown by our study, refers to the adoption by Roma of desirable attitudes and behaviors, imposed by the host country and appropriate to living in a community. However, under certain conditions, this cultural exchange can lead to social fragmentation. Return can generate social conflicts, especially if there are perceptions of inequality between those who have returned and those who have remained, both in terms of new behaviors of migrants Roma, as well as economic differences that can generate envy and marginalization from the Roma community remaining at home. In practice, we may be dealing with double discrimination and marginalization of Roma migrants, on the one hand, generated by the widespread negative stereotypes and discriminatory attitudes toward Roma (Toma, 2019) and on the other hand, a marginalization from their community, the Roma community. Our study highlighted the fact that discrimination is not a reason reported by Roma for their initial migration abroad. Only after the migration experience, by comparing the attitudes of foreigners toward them with those of Romanians in the country, could it be considered one of the reasons for secondary migration. This can be explained by the fact that Roma experience discrimination from childhood and learn to live through adaptive behaviors to discrimination.

### 5.3 Educational effects

This dimension may surprise, on the one hand, the fact that the experience abroad increases the families' interest in education, due to contact with better-structured education systems. This idea is also supported by Cherkezova (2018), who observed the relationship between Bulgarian Roma's external migratory movement and the change of their values and attitudes toward education; the Roma migrants accept more and more education as an instrument for better life achievement under certain conditions. On the other hand, there are children who, upon returning home, may encounter difficulties in adapting to the Romanian educational system, especially if they were schooled in another language or if the differences in the curriculum are significant. Although Roma, at a declarative level, value education, we find that they prefer the type of non-formal education, to the detriment of formal education. Studies (Grigore and Sarău, 2006) show that Roma prefer to educate their children themselves and involve them in various household or family activities. Our study confirms this and shows that Roma, although they have access to education, prefer to follow formal studies only up to a certain level (maximum finishing secondary school). In Romania, social policies are designed to increase the frequency of Roma in public schools, offering various monetary (scholarships, compensations) or non-monetary benefits, such as positive discrimination (special schooling places for Roma, in high schools or colleges) and the existence of school mediators in Roma communities. However, we observe from the socio-demographic data of the study that the level of education of Roma is low and very low; none of the respondents of our quantitative research have higher education. The reluctance of Roma to access these schooling places is rather related to the collective mentality of marginalization, which comes from their history (Achim, 2004) or various negative experiences lived by their family, in the past (discrimination, segregation, hate speech—FRA - European Union Agency for Fundamental Rights, 2022), but also the fear of Roma parents of distancing their children from their family of origin.

### 5.4 Effects on public policies

We note that, from the interviews with the managers and representatives of cultural institutions, the return of Roma puts pressure on local and national authorities to provide support for their reintegration, through social inclusion programs, vocational training, or educational support.

We can observe from the quantitative study that Roma did not identify as a reason for external migration, their lack of integration into Romanian society, although most of them perceive a differentiation of the state and society between them and other ethnic groups. It is rather a concept developed by social policies (Strategy of the Government of Romania for the inclusion of Romanian citizens belonging to the Roma minority for the period 2022–2027) and taken over for application by the institutions empowered to do so, including cultural institutions. This fact may suggest that the idea of integration is not an important value for Roma communities, they prefer to live in closed communities, far from other ethnic groups. On the other hand, this segregation could be a behavior learned over time, considering that it is better not to interact with authorities and other ethnic groups except in special cases, so as not to experience and reinforce discrimination and marginalization.

We believe that the push-pull factors of external migration and the effects of the Roma return to the country are interconnected, generating an amplification of the problems for which the Roma migrate. The fact that several negative effects of the Roma return to the country emerge from the interviews makes us believe that social policies aimed at the Roma should consider, along with other factors, the push-pull factors of their temporary migration. The problematic situations that the Roma face, and which determine them to migrate, remain essentially unchanged, and when they return home, moreover, in certain aspects, such as social ones (e.g., difficult reintegration or social stigmatization), they may be accentuated. Among the socio-economic effects of the Roma's return home, a discrepancy between salary expectations (increased) and the level of professional skills (low) may also be encountered, which may increase the risk of unemployment and the consumption of financial resources in a short time. This dynamic can further generate new intentions to migrate temporarily or permanently. From a cultural point of view, the exposure of Roma to new socio-cultural environments, to new relationship models, generates in them a development of the ability to recognize situations of discrimination and an increase in the unwillingness to be treated discriminatorily. The perception of discrimination becomes a substantial reason to migrate again.

The present study highlights the fact that Roma represent a vulnerable category in migration, facing discrimination and difficulties in integration in the destination countries. Recent research (Popoviciu and Tileagǎ, 2021; Ryder, 2024; Friberg, 2025) emphasizes the importance of social inclusion policies and access to services for Roma, both in countries of origin and destination, to prevent marginalization and social exclusion.

The evaluation reports show that the Romanian Government's strategy for the inclusion of Romanian citizens belonging to the Roma minority for the period 2015–2020 has only partially achieved its objectives (Government of Romania, 2022). In addition, some studies (Rostas, 2019; Rostaş and Nodis, 2022; Buhăescu-Ciucă and Ionită, 2022) suggest that effective policies for Roma integration are needed, based on in-depth research into the problems faced by Roma and how they can be involved in the development of social policies for Roma. In the absence of effective policies, the return of Roma to their country of origin can amplify the stigmatization and marginalization of Roma in society. This potential social risk situation would distance itself from the idea that the state guarantees the fundamental rights and freedoms of Romanian citizens, regardless of ethnicity, as provided for in the Romanian Constitution. It would also contravene the principle of respect for human rights, and Romania, as a European Union member state, has undertaken to respect a series of international provisions related to these aspects. In addition, the Roma population can be seen as a necessary human resource for the labor market, given that Romania also imports labor from the Asian space. All of these constitute solid arguments for streamlining social policies for the integration of the Roma.

Temporary Roma migration affects local communities in countries of origin through disruption of educational processes, administrative difficulties, precarious social conditions and risks of exclusion, but can also bring economic benefits if integrated into local development strategies. Managing these effects requires the active involvement of local authorities and more effective transnational cooperation to support the social inclusion of Roma migrants and their families.

The present article contributes to the literature on Roma migration in Europe by focusing on the distinct factors that contribute to the decision to migrate. It also adds new perspectives and dimensions to the literature on returning home decisions and experience, as well as on difficulties and opportunities during the process of reintegration.

## 6 Limitations and future research directions

Although our research provides relevant information regarding the temporary migration of Roma people, it also has certain limitations. One limitation is that the participants in the study were only from one county in Romania, so the results cannot be generalized to the entire Roma population from Romania. Future studies could expand this research to include more categories of Roma people, reflecting different patterns across various regions of Romania. Additionally, our choice of a questionnaire as a research instrument was aimed at being easy for the participants to respond to. Some of them are illiterate or have difficulty understanding or speaking, which poses challenges in data collection. We used cultural mediators and their representatives to assist, but the process was still difficult. Conducting interviews might have been more insightful in uncovering the underlying patterns in their decisions to migrate and return. As a result, we opted to gather indirect opinions from representatives of local institutions. Future research could use in-depth interviews to present a more complete picture of their migration decisions. This project is ongoing and aims to develop in several directions: on one hand, to identify solutions to reduce the external migration of Roma people because they can be an important segment of the workforce that Romania urgent needs, and on the other hand, to explore how Roma people define discrimination and how it affects their lifestyle.

## Data Availability

The raw data supporting the conclusions of this article will be made available by the authors, without undue reservation.
