# Supplementary material for: Temporary migration of Romanian Roma people to European countries
Source: Front Sociol. 2025 Jun 4;10:1577497. doi: 10.3389/fsoc.2025.1577497 (PMC12174381; doi:10.3389/fsoc.2025.1577497)
Supplement: Supplementary file 1 [file Table_1.docx]

Supplementary Material

Appendix A

Table A1. Pull-push framework. Quantitative data.

|  |  | *Direct factors (explicit reasons mentioned by Roma people)* | Indirect factors (are subtler reasons that create a favorable context for migration) |
| --- | --- | --- | --- |
| *Push factors from Romania* | Economic factors | *The primary motivations for working abroad are:*  *13.3% to get out of poverty*  *10.2 to find a job*  *67% seek a better life*  *2.5% want to earn money to build a house*  *-89.4% do not have a stable job (q10)*  *-70.3% refuse to work because the work is poorly paid (q12)*  *-11.4% refuse to work because the work is very difficult (q12)* | -47.2% consider their standard of living to be bad and very bad (q2)  -57.1% are very dissatisfied or dissatisfied with the social assistance provided by the Romanian state (q3)  -30.2% consider that the medical services provided are very bad. (q14)  -62.7% consider the lack of money to be the biggest difficulty they face in Romania  -20.2% identify finding a job as the biggest difficulty they face in Romania (q29)  -6.2% see buying a home as the biggest difficulty they face in Romania (q29)  -4.1% consider the lack of access to water or heat as the biggest difficulty they face in Romania (q29) |
|  | Socio-cultural factors | *-no sociocultural reasons such as discrimination*  *-only 6% report that their work colleagues have negative attitudes towards their ethnicity (q12)* | -37.9% believe that the state takes no action to facilitate access to education for Roma people (q4)  -4.5% describe their relationships with their neighbors as bad or very bad (q9)  -39.4% are very dissatisfied with their lives. (q15)  -48.4% feel that their rights are not respected (q28)  -5.3% consider the lack of respect from others to be the biggest difficulty they face in Romania (q29)  -49.3% feel they are largely judged based solely on their ethnicity (q31)  44.2% report feeling disadvantaged compared to other ethnic groups (q32) |
| *Pull factors to other countries* | Economic factors | -72.4% opportunity to make money (q18)  14.5% where they migrate, they live in relatives' home (q23)  3.7% where they migrate, live in houses provided by their employer |  |
|  | Socio-cultural factors | 18.3% the way people treat them (q18)  4.3% the climate (q18)  71.3% say they feel treated better in other countries (q24)  76.5% have fewer difficulties than in Romania (q25)  67.4% foreigners are more willing than Romanians to help them (q26) | 41.9% go abroad with their spouse or other relatives (q21)  21.5% go abroad with friends (q21) |
| *Push factors from other countries (for returning)* | Economic factors | 4.4% contract expiration as the reason for return (q21)  66.8% where they migrate live in rented spaces (q23)  8% where they migrate they live in uninhabited buildings (q23)  4.2% where they migrate they live in their vehicles or caravan (q23)  0.4% where they migrate live on the street/park (q23) |  |
|  | Socio-cultural factors | -10.5% say they feel treated worse in other countries (q24)  -9.1% have more difficulties than in Romania (q25)  -13.7% foreigners are less willing than Romanians to help them (q26) |  |
| *Pull factors to Romania (for returning)* | Economic factors | There were no specified |  |
|  | Socio-cultural factors | 74.1% return for families (q21)  12.4% return because they have a house in Romania, they feel at home here (q21)  2.7% return because having friends here (q21) |  |

Appendix B

Table B1. Pull-push framework. Qualitative data. Categorization matrix

| Factors | Sub-categories of factors | What is it? | Quotes from managers of socio-cultural institutions |
| --- | --- | --- | --- |
| *Push factors from Romania* | Economic factors | Poverty  Low-paid jobs  Unstable jobs  Low standard of living  Lack of jobs | "because of financial deprivation”  "They are people without income, with a poor education, who have difficulty finding a job due to school absenteeism"  "the proportion of Roma in employment is significantly low"  "a large proportion of them live on social benefits without education and without the possibility to obtain a qualification"  "lack of job offer on the job market" |
|  | Socio-cultural factors | Discrimination  The nomadic tradition of the Roma population  Lack of social integration programs | "members of the Roma community are still victims of discrimination, which stops them from being properly educated and integrated into society"  "the existence of a tradition of migrating abroad"  "the desire for the new, it is known that they were and still are nomads"  "because they were originally nomads and they want to know new societies"  "the state carries out few actions because it focuses more on helping them through financial measures instead of focusing on their integration" |
| *Pull factors to other countries* | Economic factors | The opportunity to earn more money  Well paid jobs  Satisfactory standard of living  Recommendations of the country by acquaintances, friends | "the potential for faster and easier financial gain"  "the dream of having substantial income”  "the desire to get rich”  "believe they can get some money much easier than in Romania"  "the higher standard of living is the reason for the migration of the Roma population to Western countries"  "Because there are already large Roma communities there” |
|  | Socio-cultural factors | Non-discriminatory attitude  Help received from the citizens of the host country  Possibility to learn the language of the host country more easily  Permissive legislation with immigrants  Climate  Cultural diversity  Development | "Overcoming stereotypes regarding ethnicity by the inhabitants of Spain, Italy"  "the languages spoken there are easy to learn"  "because of linguistic similarities"  "there are countries that have permissive legislation towards migrants"  "they are more easily accepted by society, there are countries with a relaxed social level which is good for children"  "the climate they are used to, from the point of view of origin"  " for new development opportunities that they don't have in Romania" |
| *Push factors from other countries (for returning)* | Economic factors | The gap between expectations and reality  Lack of integration into the labor market in the host country | "the difficulties faced by Roma in terms of inclusion in the labor market"  "the strictness of society abroad"  "the strict work schedule abroad" |
|  | Socio-cultural factors | Lack of social integration in the host country  Lack of participation in local socio-cultural events  Avoiding punishments | "because they are not accepted most of the time, due to their different behavior"  "because of the problems that some of them cause from a social point of view"  "the rigidity of the societies they migrated to"  "The lack of adaptation to the social environment in European countries"  "they often break the law of the respective country and return home to escape the rigors of the law" |
| *Pull factors to Romania (for returning)* | Economic factors | Investing in Romania their earned money | “accumulating financial resources that they want to invest at home” |
|  | Socio-cultural factors | Family  Birthplaces  Traditions and customs  More relaxed Romanian legislation  Possibility of social affirmation (social validation from the community) | "the family and the environment they come from are an important factor in the return of Roma to their country of origin, the fact that they are deeply connected to everything that culture and tradition mean"  "the Romanian national space is attractive and hospitable and Roma traditions are anchored in customs and habits that can only be expressed in Romania"  "Romanian legislation that is not as demanding as that of the countries where they migrate"  "the desire to assert themselves towards those who remain at home" |

Appendix C

Table C1. - The effects of Roma return migration. Qualitative data. Categorization matrix.

| Sub-categories of effects | What is it? | Quotes from managers of socio-cultural institutions |
| --- | --- | --- |
| Positive effects | economic growth | "They bring money into the country that will be spent here"  "they have invested, opened businesses, and provided jobs to those in their community"  "When they come back they can increase their purchasing power for a while"  "raising the standard of living"  "they bring money into the country"  "opening a business" |
|  | cultural exchange | "the emergence of customs specific to the countries from which they returned"  "moving from begging to the villa" |
|  | awareness of the role of education | „if they've been to school when they come back, they equalize their studies from abroad” |
| Negative effects | increasing crime rate | "if only the Roma who created social problems return, then the effects are negative"  "the number of thefts increases"  "when they return they commit crimes" |
|  | increasing unemployment | "the emergence of an increasing number of unemployed people"  "the unemployment rate is increasing"  "the inability to find a job"  "constant obstacles in accessing the labor market, due to poor training and unequal access to quality education"  "the labor market cannot absorb them"  "an imbalance in society" |
|  | difficulties in children's education | "the general difficulty of children's readjustment to school" |
|  | the negative image of Romania | "they are confused with Romanians"  "they have a bad reputation and don’t honor our country"  "problems related to the image of the country, mostly they appear in the media space because of the crimes they committed"  "they spoil the image of the country – abroad, they are begging and stealing"  "decrease in foreigners' trust in Romanian citizens" |
